# Modeling the Effect of Cold Stratification on Seed Germination Performance of *Rudbeckia fulgida* Aiton Using Response Surface Methodology (RSM)

**DOI:** 10.3390/plants15020220

**Published:** 2026-01-10

**Authors:** Türker Oğuztürk, Cem Alparslan, Merve Sipahi, Gülcay Ercan Oğuztürk, Ece Nur Topaloğlu, Şenol Bayraktar, Turan Yüksek

**Affiliations:** 1 Department of Landscape Architecture, Faculty of Engineering and Architecture, Recep Tayyip Erdoğan University, Rize 53020, Türkiye; turker.oguzturk@erdogan.edu.tr (T.O.); ecenur_topaloglu22@erdogan.edu.tr (E.N.T.); turan.yuksek@erdogan.edu.tr (T.Y.); 2 Department of Mechanical Engineering, Faculty of Engineering and Architecture, Recep Tayyip Erdoğan University, Rize 53020, Türkiye; cem.alparslan@erdogan.edu.tr (C.A.); senol.bayraktar@erdogan.edu.tr (Ş.B.); 3 Department of Interior Architecture and Environmental Design, Faculty of Architecture, Design and Fine Arts, Osmaniye Korkut Ata University, Osmaniye 80000, Türkiye; mervesipahi@osmaniye.edu.tr

**Keywords:** *Rudbeckia fulgida*, cold stratification, dormancy, seed germination, RSM

## Abstract

This study investigates the influence of varying cold stratification durations (0–165 days) on the germination performance and early seedling development of *Rudbeckia fulgida*. Seeds were divided into 11 groups at 15-day intervals, using a total of 1320 seeds. For each stratification duration, an equivalent number of seeds stored at room temperature served as non-stratified controls. Results demonstrated a clear and significant increase in germination percentage with longer stratification periods (Kruskal–Wallis, H = 57.03, *p* < 0.001), with the highest germination observed at 135 and 165 days (96.7%). In contrast, seeds kept at room temperature exhibited low and inconsistent germination. Strong positive correlations were detected between stratification duration and both germination percentage (r = 0.914) and post-stratification seed weight (r = 0.419). Furthermore, a Response Surface Methodology (RSM) model was developed to predict germination behavior, achieving an exceptionally high 99% predictive accuracy. The RSM analysis confirmed that cold stratification duration is the dominant factor shaping germination responses in *Rudbeckia fulgida* Aiton. Overall, the study demonstrates that cold stratification is essential for breaking seed dormancy in *R. fulgida*, substantially improving propagation efficiency and offering valuable insights for nursery production, landscape practices, and restoration ecology.

## 1. Introduction

Seed germination represents a critical transition from the quiescent embryonic state to autotrophic growth, and its timing plays a decisive role in plant survival, fitness, and long-term population persistence [1,2]. Seeds must precisely interpret environmental cues—including temperature, moisture, and light—to ensure that emergence aligns with periods favorable for seedling establishment [3]. In temperate ecosystems, germination timing is tightly controlled by seed dormancy, an adaptive trait enabling seeds to delay germination until environmental conditions become optimal [4,5]. Dormancy thereby buffers populations against temporal heterogeneity and supports species continuity under fluctuating climates [6,7,8]. Studies highlight that dormancy patterns are influenced both by long-term evolutionary pressures and short-term environmental conditions experienced during seed maturation, storage, and soil persistence [9,10,11]. Seed dormancy is governed by a complex interplay of environmental signals and endogenous hormonal pathways. Temperature acts as the principal regulator of annual dormancy cycling in the soil seed bank, modulating sensitivity to secondary signals such as light and nitrate [3]. Maternal environmental effects—particularly low temperatures during seed development—increase dormancy depth, illustrating the ecological and evolutionary plasticity of seed responses [12,13]. Seeds integrate seasonal temperature and moisture patterns through the “temporal window” and “spatial window” mechanisms, in which stratification determines when seeds become competent to germinate, while microsite-level cues determine whether germination proceeds [3,14]. Molecularly, the antagonistic interaction between abscisic acid (ABA) and gibberellins (GA) remains the core regulatory axis of dormancy and germination. ABA maintains dormancy by repressing embryo growth, while GA promotes germination through activation of genes such as *GA3ox1* and degradation of DELLA proteins [9,15]. Cold stratification triggers GA biosynthesis, ABA catabolism, and extensive transcriptomic remodeling, facilitating radicle protrusion [8,16]. Recently, brassinosteroids (BRs) have emerged as additional regulators capable of promoting germination by counteracting ABA signaling. Cold exposure induces the expression of BR pathway genes (*DWF4*, *DET2*) and cold-responsive transcription factors (*CBF1*, *CBF2*), forming a BR–ABA–GA interaction network essential for stratification-mediated dormancy release [17]. Cold stratification—typically defined as imbibition of seeds at low temperatures (~4 °C)—is one of the most widespread ecological and horticultural methods for alleviating physiological dormancy [18,19,20]. Stratification-mediated dormancy release involves hormonal rebalancing, endosperm weakening, ROS signaling, and expression of dormancy-associated genes including *DOG1* [4,16]. Transcriptomic studies show that stratification modifies gene networks associated with cell wall remodeling, ABA catabolism, GA biosynthesis, and cold response pathways [16]. However, it should be noted that bulk transcriptomic approaches provide an averaged signal across multiple seed tissues and cell types and therefore do not capture tissue-specific or cell-specific regulatory dynamics during germination. This artificial simulation of winter conditions promotes dormancy release through increased water uptake, enhanced oxygen diffusion, weakening of seed coverings, and improved synchronization of hormonal signaling cascades and gene expression responses associated with dormancy release [21,22,23,24]. Cold stratification is therefore understood not as a genetic reprogramming process, but as a preparatory phase that synchronizes physiological and hormonal cascades while preventing premature depletion of endogenous seed reserves prior to germination. Numerous species demonstrate significant improvements in germination following stratification. For example, *Primula beesiana*, *Aster tripolium*, and *Triglochin maritimum* exhibit maximized germination at 30–60 days of cold treatment [25]. Likewise, *Pinus koraiensis* displays a strong positive correlation between stratification duration and germination percentage [26]. These examples emphasize the biological relevance of stratification length as a determinant of germination success. Seed germination represents a complex but tightly coordinated biological process governed by spatially and temporally synchronized physiological mechanisms, including metabolic activation, maintenance of embryonic viability, and environmental signal perception. Pretreatments such as temperature, moisture content, and stratification are among the most critical determinants of this process and often interact with each other to produce nonlinear responses. Therefore, statistical approaches that can evaluate the simultaneous effects of multiple factors are needed in plant physiology research. Response Surface Methodology (RSM) is increasingly used due to its high-accuracy representation of this multifactorial structure, which is common in biological systems, in modeling and optimization processes [27,28]. Indeed, in recent years, RSM has been successfully applied in plant sciences in engineering applications parallel to rooting efficiency, urban microclimate relationships, and material-biological interaction processes [27,29,30,31,32]. This multidimensional analysis capacity of RSM provides a methodological advantage, particularly in studies where multiple environmental and physiological variables, such as temperature, stratification time, and weight differences, exert simultaneous effects on germination. In this context, this study evaluated how stratification, a frequently applied physiological pretreatment in seed germination, shapes the germination response along with temperature and moisture dynamics using two different systems. In the first system, seeds were stratified for varying lengths of time; in the second system, control conditions were created without any pretreatment. In both systems, key environmental variables such as temperature, weight change (pre-post gram difference), and stratification time were measured, with germination percentage used as the response variable. RSM was applied to quantify the effects of these variables on germination, compare the germination performance of stratified and non-stratified systems, and determine under which conditions germination is optimized. Thus, the study provides a comprehensive model of the germination process by evaluating the physiological effects of stratification not only at the single factor level but also through multidimensional responses created by temperature and moisture (two-way interactions). This study focuses on controlled dormancy release and predictive modeling rather than natural ecosystem-level stratification dynamics. *Rudbeckia fulgida* Aiton belongs to the family Asteraceae, which includes ecologically and horticulturally important taxa [33,34] and is native to eastern North America, where it occurs in a variety of habitats from temperate grasslands to calcareous wetlands [35]. The species is particularly characteristic of localized wetland systems in Alabama, Florida, and Georgia, where it is considered a regionally rare taxon [36]. Prior research on the species has focused on population viability, pollinator community diversity, essential oil chemistry, and general ecological distribution rather than detailed germination physiology [33,34,37]. Although several studies indicate the presence of physiological dormancy in Rudbeckia seeds, species-specific stratification requirements vary across habitats and seed sources [38], underscoring the need for quantitative germination analyses. Morphologically, *R. fulgida* exhibits hairy, serrated, and variably lobed leaves, with yellow to dark purple disk florets and orange to yellow ray florets arranged on a prominent conical receptacle [39]. Beyond its ecological role, *R. fulgida* holds considerable practical value. In landscape architecture, the species is valued for ornamental aesthetics, drought tolerance, and extended flowering performance [40]. Ethnobotanical records indicate its traditional medicinal use by Native American communities for treating earaches, wounds, venereal diseases, and various inflammatory conditions [33,41]. Recent experimental studies report that extracts of *R. fulgida* exhibit anticancer properties by inhibiting cellular proliferation under controlled laboratory conditions [42]. These multifunctional attributes underscore the species’ relevance across ecological restoration, horticultural production, and biomedical interest. Despite its ecological and practical significance, propagation of *R. fulgida* from seed is challenging due to the presence of physiological dormancy. Its seeds exhibit strong resistance to heat, cold, and pathogens, combined with high water permeability influenced by parental genetic traits [43]. These characteristics complicate direct germination and hinder large-scale seedling production. Cold stratification has been reported as one of the most effective methods for breaking dormancy in this genus [44], yet species-specific guidelines—particularly concerning optimal stratification duration—remain insufficiently documented. Existing literature on *Rudbeckia* primarily addresses habitat, taxonomy, essential oil composition, and population viability [33,38,45], while quantitative germination studies remain remarkably limited. Given that germination is shaped by complex interactions among environmental conditions, seed physiological traits, and dormancy-regulating mechanisms, the response of *Rudbeckia fulgida* to stratification duration represents a multidimensional biological process. Traditional statistical approaches often fail to fully capture such nonlinear and interactive effects. The novelty of this study does not lie in the mathematical method itself, but in its application to *Rudbeckia fulgida* using a fine-resolution stratification design (0–165 days at 15-day intervals) and in the development of a highly accurate predictive model for dormancy release. To our knowledge, such a high-resolution stratification framework combined with response surface modeling has not been previously reported for this taxon. Therefore, in this study we employed Response Surface Methodology (RSM), a quantitative modeling framework capable of describing curvature, interaction terms, and optimal response regions through second-order polynomial models. Using germination data obtained across eleven stratification durations ranging from 0 to 165 days at 15-day intervals, the RSM model demonstrated excellent predictive performance, achieving 99% accuracy. The use of 15-day interval stratification, which has not been previously reported for this taxon, constitutes the principal novelty and scientific contribution of this work. By identifying the most effective chilling duration and providing a reproducible predictive model for germination outcomes, this study offers practical insights for seed propagation, nursery production, ecological restoration, and biodiversity-oriented landscape applications.

## 2. Materials and Methods

### 2.1. Material

**Figure 1:**
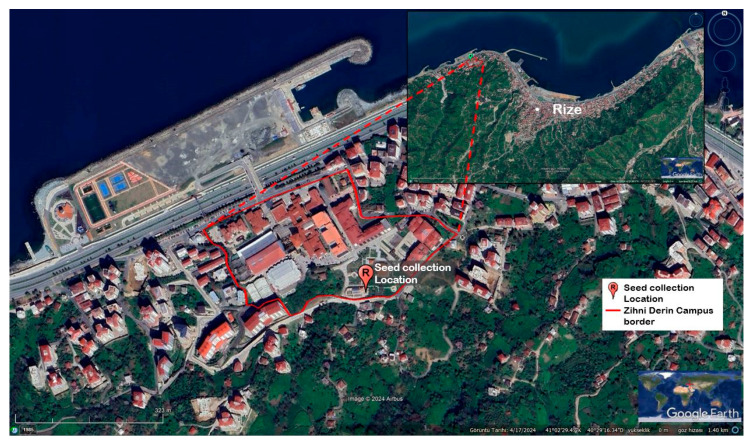
Satellite image showing the location of the mother plant from which *Rudbeckia fulgida* Aiton seeds were collected. The red marker indicates the seed collection site within the boundaries of Recep Tayyip Erdoğan University Zihni Derin Campus (Rize, Türkiye).

The biological material used in this study consisted of mature seeds of *Rudbeckia fulgida* Aiton. Healthy and fully developed seeds were collected on 23 October 2024 from a single individual located in Fener District, Rize, Türkiye, within the Zihni Derin Campus of Recep Tayyip Erdoğan University (41°2′11.12″ N latitude, 40°29′43.60″ E longitude) (Figure 1). The seeds were manually harvested from the inflorescences of a single mother plant grown outdoors in the greenhouse area of Recep Tayyip Erdoğan University (RTEU). The geographic coordinates of the mother plant were recorded using a GPS device. Only well-filled, healthy, and undamaged seeds from the same aspect and flowering position were collected. After collection, the seeds were transported to the laboratory, air-dried at room temperature, cleaned to remove foreign materials, and stored in a dry and dark environment until the stratification procedure began.

### 2.2. Experimental Design

Mature and healthy *Rudbeckia fulgida* seeds collected from the specified location were divided into eleven treatment groups corresponding to different cold stratification durations: Control (0 days), 15, 30, 45, 60, 75, 90, 105, 120, 135 and 165 days of cold exposure. Each stratification interval consisted of six replicates, with 10 seeds per replicate, totaling 60 seeds per treatment. Across the eleven stratification treatments, a total of 660 seeds were used.

In addition to the stratified seeds, an equal number of seeds were kept at room temperature throughout the study to serve as a non-stratified control group. These seeds were sown simultaneously with the seeds removed from cold stratification at each interval. Each stratification group was labeled with an alphabetical code: A = Control (0 days), B = 15 days, C = 30 days, D = 45 days, E = 60 days, F = 75 days, G = 90 days, H = 105 days, I = 120 days, J = 135 days, and K = 165 days. In addition, the non-stratified seeds that were kept at room temperature and sown simultaneously with each chilled group were labeled as A2, B2, C2, D2, E2, F2, G2, H2, I2, J2, and K2. A total of 1320 seeds were used throughout the entire study (Table 1).

Germination was conducted under controlled room conditions. Ambient temperature was monitored daily for 15 days following sowing, and the mean temperature during the germination period was used in subsequent statistical and RSM analyses. Non-stratified seeds maintained continuously under the same room-temperature conditions and sown simultaneously with each stratified group were used as the experimental control. Before initiating the treatments, all seeds were visually inspected, cleaned of debris, and stored under dry conditions. Each seed group was then placed into labeled perforated bags to maintain sample identity throughout the process. Cold stratification was performed in plastic containers filled with previously sterilized soil medium (university nursery-produced, Recep Tayyip Erdoğan University, Rize, Türkiye). A thin layer of this soil was spread at the base of each container, after which the seed bags were positioned on the surface and covered with an additional layer (approximately 1 cm) of the same moistened substrate. This setup ensured constant contact between the seeds and the humid medium without allowing waterlogging.

Soil moisture was initially adjusted to an optimal level and periodically checked throughout the stratification phase. Sterile distilled water (laboratory-prepared, Recep Tayyip Erdoğan University, Rize, Türkiye) was added when necessary to maintain consistent humidity. All containers were stored in a refrigerator (Arçelik, İstanbul, Türkiye) maintained at 4 ± 0.5 °C, mimicking natural winter conditions. The boxes were loosely covered to prevent excessive moisture loss while still allowing sufficient air exchange. Throughout the chilling period, the containers were routinely inspected to detect any fungal contamination; any infested material was removed immediately to protect the remaining seeds. Additional seed batches were also prepared separately to serve as backups in case replacement was required during the study. During the entire stratification period, an identical set of seed batches was stored under constant room temperature conditions and was not exposed to chilling treatments, ensuring a parallel control group for evaluating baseline germination capacity.

### 2.3. Germination Procedure

The germination phase of the experiment began on 31 January 2025. Once each cold stratification period was completed, the seed batches were removed from their mesh bags and carefully transferred to germination units. At each sowing time, seeds kept at room temperature were planted simultaneously with the chilled seeds to ensure comparable germination measurements across all groups. Every seed was placed into small vials containing pre-sterilized soil, and after positioning them, a thin layer of the same substrate was gently spread over the surface. To improve aeration and prevent surface crusting, a uniform layer of fine perlite (İyibahçe, İstanbul, Türkiye) was added on top of the covering soil.

All germination tests were carried out inside sterile, lidded plastic containers kept under controlled environmental conditions. Germination was carried out in a controlled room environment, where ambient temperature was monitored daily for 15 days following sowing. The mean temperature during the germination period was calculated and used as an input variable in subsequent statistical analyses and Response Surface Methodology (RSM) modeling. Seeds were sown in round plastic sterile boxes (Fıratmed, Ankara, Türkiye), 90 mm in diameter, 40 mm in height, and germination progress was monitored daily for 30 days. Germination was considered successful when seedlings reached approximately 1 cm in height, indicating stable radicle and hypocotyl development.

### 2.4. Data Sets

For each stratification duration, germination success was calculated as the percentage of seeds that germinated in relation to the total number of seeds in each replicate. Each stratification treatment consisted of six replicates, and germination percentages were computed separately for every replicate. Following sowing, the temperature of the experimental environment was measured daily for 15 consecutive days, and the mean of these measurements was used as the temperature input variable during the stratification modeling phase.

For all seed groups, pre-stratification seed weights were measured using a precision balance (Yıldırımtartı, İstanbul, Türkiye) with an accuracy of 0.001 g. After completing their respective stratification periods, the seeds were individually cleaned and reweighed on the same balance prior to sowing. The difference between the two measurements represented the change in seed mass (in grams) during the stratification process.

All collected data—including germination percentages, temperature means, and pre- and post-stratification seed weights—were used to assess how different cold stratification durations influenced the germination behavior of *Rudbeckia fulgida*. Post-stratification seed weight refers to the seed mass measured immediately after cold stratification and prior to germination, reflecting water uptake during imbibition.

### 2.5. Data Analysis

All statistical analyses were performed using IBM SPSS Statistics 29 (IBM Corp., Armonk, NY, USA). Since several treatment groups violated the assumptions of normality and homogeneity of variances, germination percentages were compared using the non-parametric Kruskal–Wallis test. When significant overall differences were detected (*p* < 0.05), pairwise group comparisons were conducted using Bonferroni-adjusted post hoc tests to identify which stratification durations differed from one another.

In addition to group-based analyses, Spearman’s rank correlation coefficients were calculated to evaluate the relationships among cold stratification duration, temperature conditions, pre-stratification seed weight, post-stratification seed weight, and weight change. This non-parametric approach was selected because it does not require normally distributed data and is appropriate for examining monotonic biological associations.

This combined analytical framework—integrating non-parametric group comparisons with correlation-based assessments—provided a robust evaluation of dormancy-breaking responses in *Rudbeckia fulgida*. Overall, the statistical design enabled a comprehensive interpretation of how cold stratification duration influences germination performance, seed physiology, and early seedling development, offering valuable insights for propagation strategies and practical horticultural applications.

### 2.6. Statistical Analysis: Response Surface Methodology (RSM)

In this study, all measurements from germination experiments conducted in two different experimental systems (with and without stratification) were systematically recorded and organized in preparation for the analysis process. Standard data collection forms were used to minimize potential experimental errors in the measurements, and all temperature, weight, and time values were transferred to Excel (Office 365, Microsoft Corp., Redmond, WA, USA) spreadsheets via digital recording systems. Care was taken to maintain constant environmental variables (ambient temperature, relative humidity, and light intensity) throughout the experiment, improving data reliability. To ensure the accuracy of the parameters used in the measurements, repeat measurements were taken at regular intervals, and data consistency was checked. This preprocessing process aimed to reduce the impact of systematic errors that could arise from measurement instruments and data entry and to ensure homogeneous data analysis.

In further data analysis, Response Surface Methodology (RSM) was used to model and optimize the factors influencing germination percentage. RSM is a powerful experimental design and analysis approach that allows for the evaluation of relationships between the dependent variable and multiple independent variables, including both linear and nonlinear (squared and interaction) components. RSM has been successfully applied in both plant physiology and engineering contexts in the literature [46,47]. RSM was chosen in this study because germination percentage is simultaneously affected by multiple factors such as stratification time, temperature, and weight difference, and because nonlinear interactions are possible between these factors. Furthermore, the statistical significance of input variables over output variables was determined using ANOVA (Analysis of Variance) at a 95% confidence level and a *p* < 0.05 criterion. Optimization and ANOVA studies were conducted using Minitab 17.0 (Minitab LLC, Pennsylvania State University, USA) [48,49]. In the RSM models created within this scope, the dependent variable was germination percentage, and the independent variables were stratification time (A, days), ambient temperature (B, °C), and weight difference (C, g). Models were created separately for stratified and non-stratified systems. In both models, the main effects (A, B, and C), two-way interaction terms (AB, AC, and BC), and quadratic terms (A^2^, B^2^, and C^2^) were evaluated using a second-order multiple regression approach. The quadratic mathematical model to be used in this analysis is given below (Equation (1)). Here, a_0_, bi, bij, bii, and i, j, k = 1, 2, 3…n represent the regression coefficients of the model, Xi and Xj represent the descriptive factors, and Y represents the experimental outcome (germination percentage) in Equation (1).

(1) $$\begin{array}{cccc} & Y=a_{0}+\sum_{i=1}^{k}\sum b_{i}X_{i}+\sum_{ij}^{k}b_{ij}X_{ij}+\sum_{i=1}^{k}b_{ii}X_{i}^{2} & \; & \end{array}$$

Model validity was assessed using ANOVA results, R^2^, adjusted R^2^ (Adj-R^2^), predictive R^2^ (Pred-R^2^), AIC, BIC, and PRESS values. Residual analyses were conducted using normal probability plots, Versus Fits, and Versus Order plots to verify that the assumptions of normal distribution, homogeneity of variance, and independence were met. The mathematical models developed for both systems were subjected to validation analyses by comparing them with experimental data, and the agreement between predicted and observed values was examined. This validation process was conducted to assess the reliability of the models and their predictive performance against factor variations. Finally, optimal conditions for maximum germination were determined using RSM outputs, and the different response profiles of the systems with and without folding were compared.

## 3. Results

### 3.1. Germination Success

**Figure 2:**
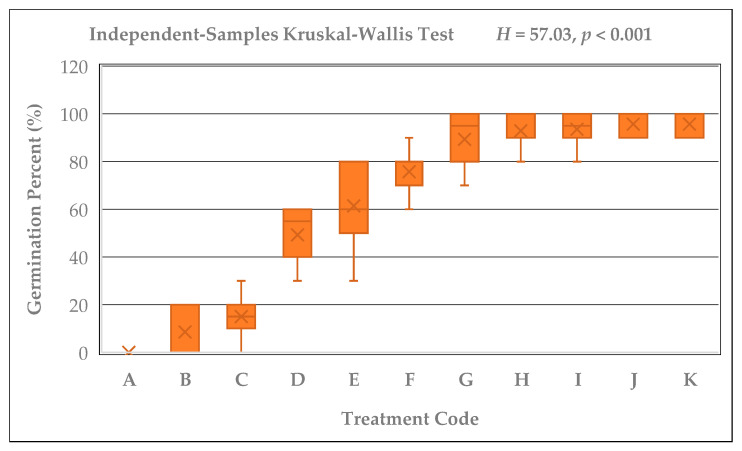
Kruskal–Wallis boxplot illustrating the germination percentages of *Rudbeckia fulgida* seeds across cold stratification durations (A–K; 0–165 days) (H = 57.03, *p* < 0.001). Germination rates show a consistent and pronounced increase with longer stratification periods.

**Figure 3:**
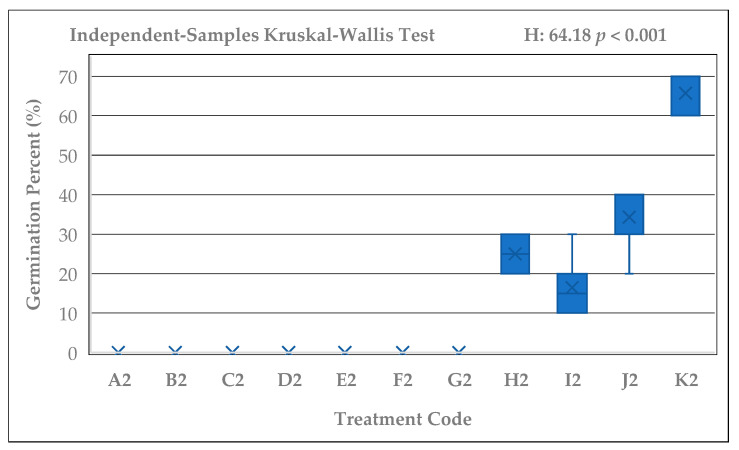
Germination percentages of *Rudbeckia fulgida* seeds kept at room temperature for the same durations (A2–K2). No germination was observed in the early-period groups, while only low to moderate germination occurred in the long-duration groups (H2–K2) (H = 64.18, *p* < 0.001).

Significant differences were detected among the germination percentages of *Rudbeckia fulgida* seeds subjected to different cold stratification durations (A–K; 0–165 days) (Kruskal–Wallis test; H = 57.03, *p* < 0.001). Likewise, the control seeds that were kept at room temperature for the same durations without cold stratification (A2–K2) also showed significant differences within their group (Kruskal–Wallis; H = 64.18, *p* < 0.001). Germination percentages exhibited a clear and consistent increase as the stratification period lengthened. The lowest germination was observed in group A (0%), whereas groups J and K, which received 135 and 165 days of stratification, exhibited the highest average germination rate (96.67%). In addition, consistently high germination percentages (≥90%) were recorded only in groups G, H, I, J, and K, corresponding to stratification durations of 90 days or longer. A rising trend in germination percentage was also evident graphically among the stratified seed groups (Figure 2). While germination was nearly absent in groups A and B, a marked increase emerged after 60 days, and groups exposed to 105, 120, 135, and 165 days showed 90–100% germination. In contrast, non-stratified seeds exhibited no germination in the A2–G2 groups, and only limited and irregular germination was observed in H2, I2, J2, and K2 (Figure 3). Pairwise comparisons with Bonferroni adjustment revealed that the differences between early-period groups (A–C) and late-period groups (G–K) were largely significant (Adj. *p* < 0.05).

### 3.2. Pairwise Comparison Network

**Figure 4:**
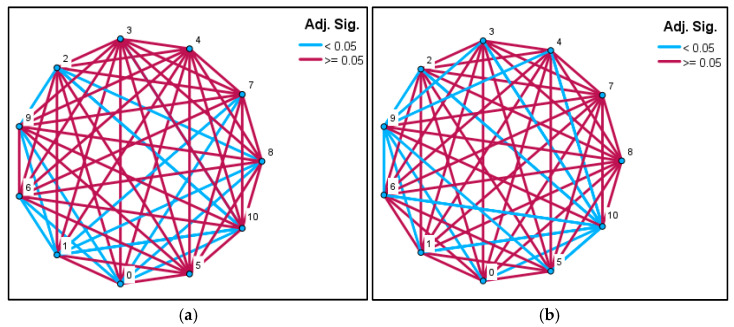
Network diagrams showing Bonferroni-adjusted pairwise comparison results for different cold stratification durations in *Rudbeckia fulgida* seeds. (**a**) Pairwise comparisons based on mean rank values for seeds subjected to cold stratification. (**b**) Pairwise comparisons based on mean rank values for seeds kept at room temperature for the same durations. Blue lines indicate statistically significant differences (Adj. *p* < 0.05), while burgundy lines represent non-significant comparisons (Adj. *p* ≥ 0.05). Node numbers correspond to stratification durations as follows: 0 = 0 days, 1 = 15 days, 2 = 30 days, 3 = 45 days, 4 = 60 days, 5 = 75 days, 6 = 90 days, 7 = 105 days, 8 = 120 days, 9 = 135 days and 10 = 165 days.

Examination of the pairwise comparison network for the cold-stratified groups shows numerous significant differences between the short-duration stratification groups (A–C) and the long-duration treatments (F–K) (Adj. *p* < 0.05; blue lines). In particular, groups A, B, and C were statistically distinct from nearly all late-period groups, whereas the longest stratification treatments (H, I, J, K) did not differ significantly from one another (Adj. *p* ≥ 0.05; burgundy lines). This pattern indicates that germination performance improves markedly with increasing stratification duration, and that extended chilling durations converge toward similar germination levels (Figure 4a). For the non-stratified seeds, the pairwise comparison network reveals no significant differences among most short-duration groups (A2–G2), largely because germination did not occur in these treatments (Adj. *p* ≥ 0.05). In contrast, the long-duration room-temperature groups (H2, I2, J2, K2) produced several significant differences (Adj. *p* < 0.05; blue lines). Notably, the K2 group, which showed the highest germination in the non-stratified set, was statistically distinct from all other groups. This pattern suggests that a limited degree of spontaneous dormancy release may occur in seeds kept at room temperature for extended periods; however, the effect is irregular and comparatively weak (Figure 4b).

### 3.3. Correlation Analysis

**Figure 5:**
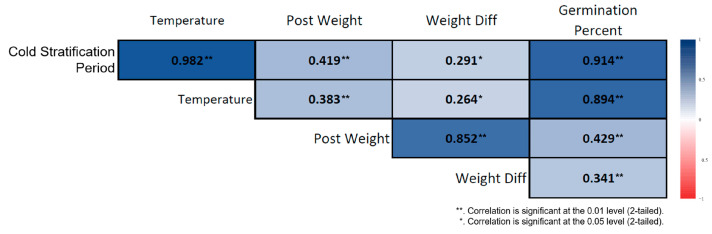
Correlation heatmap for cold-stratified *Rudbeckia fulgida* seeds. Significant relationships were observed among stratification duration, germination percentage, and post-germination biometric traits. Darker-colored cells indicate stronger correlation coefficients (*p* < 0.01, **; *p* < 0.05, *).

**Figure 6:**
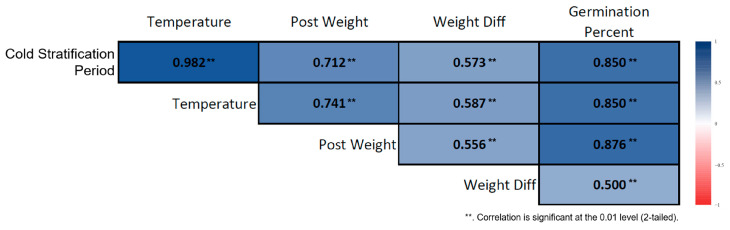
Correlation heatmap for non-stratified *Rudbeckia fulgida* seeds. The heatmap illustrates the relationships between ambient temperature, germination percentage, and post-germination biometric seed traits in seeds that were not subjected to cold stratification. Darker-colored cells represent stronger correlation coefficients (*p* < 0.01, **).

Spearman correlation analysis conducted on the cold-stratified samples revealed strong and significant associations among the variables involved in the germination process. Germination percentage increased almost linearly as the stratification duration lengthened. Stratification duration showed a very strong positive correlation with germination percentage (ρ = 0.914, *p* < 0.01) (Figure 5). Ambient temperature was also strongly and positively correlated with germination percentage (ρ = 0.894, *p* < 0.01). In seeds subjected to long-term stratification, post-stratification seed weight increased, and stratification duration was positively associated with seed weight (ρ = 0.419, *p* < 0.01). Although weight difference was sensitive to stratification duration, its overall effect was limited (ρ = 0.291, *p* < 0.05). In the non-stratified seeds, the correlation structure differed markedly. Germination showed a slight increase with prolonged room-temperature storage, but this increase was irregular and biologically weak. Nevertheless, a strong positive correlation was found between storage duration and germination percentage (ρ = 0.850, *p* < 0.01) (Figure 6). Similarly to the stratified seeds, germination percentage in non-stratified seeds exhibited strong positive correlations with ambient temperature (ρ = 0.982, *p* < 0.01) and seed weight (ρ = 0.573, *p* < 0.01).

The correlation structure of cold-stratified seeds demonstrates that the germination process of *Rudbeckia fulgida* is strongly dependent on stratification duration. As stratification length increased, both germination percentage and physiological development indicators (PostWeight, Weight_Diff) increased markedly. This confirms that cold stratification is an essential step in breaking dormancy in *R. fulgida* seeds. Although non-stratified seeds also showed high correlation coefficients, their overall germination rates were very low, indicating that *R. fulgida* seeds are not physiologically ready to germinate without a cold stratification phase.

### 3.4. RSM Findings Regarding Germination Percentage in the Non-Straightening Group

The RSM experimental design for the non-stratified system demonstrated a strong statistical explanation of the key factors determining germination percentage. The model’s coefficients of determination and adjusted coefficients of determination were calculated as R^2^ = 85.04% and Adj-R^2^ = 78.14%, respectively, indicating that a substantial proportion of the variability in germination percentage was explained by the selected independent variables. The relatively high Adj-R^2^ value confirms that the model maintains strong explanatory power after accounting for the number of predictors, suggesting that overfitting was minimized and that the fitted response surface provides a reliable representation of the germination response to time-related variable, temperature, and weight-related variables. These values demonstrated that the selected experimental variables could analyze germination behavior with high accuracy (Table 2). Consequently, the statistical significance of the model was found to be quite high (F = 12.32; *p* < 0.001). The contribution of linear terms to the model was 71.52%, and the temperature factor, in particular, had a dominant effect on germination (F = 19.68; *p* = 0.001). However, as expected, stratification time did not have a significant effect on the non-stratified group (*p* = 0.990). While the weight difference alone did not cause a significant change in germination (*p* = 0.249), it made a limited contribution to the model. The total contribution of the squared terms was 11.74%, and the quadratic term for temperature was found to be significant (F = 11.52; *p* = 0.005). This suggests that temperature has a nonlinear, curvilinear effect on germination, indicating an optimal range. In contrast, the squared term for the weight difference was not statistically significant (*p* = 0.829). All two-way interaction terms (B × C, B × A) were found to be insignificant and it was observed that these interactions did not create a significant synergistic effect on germination (Table 3).

The model’s quadratic regression equation (Equation (2)) clearly demonstrates that temperature exerts both linear and curvilinear effects on germination percentage. The positive temperature coefficient and the statistically significant squared term in the equation indicate that the germination response increases with increasing temperature up to a certain optimum range and exhibits a plateau-like behavior above this range. Although the weight difference term is represented by a negative coefficient in the equation, the magnitude and significance of the coefficient indicate that this factor exerts a limited, secondary pressure on germination. The near-zero and insignificant doubling time coefficient is an expected finding due to the nature of this group (0 days in all samples). The insignificance of the interaction terms (B × C, B × A) despite their inclusion in the equation indicates that temperature and weight difference do not create a synergy that works together on germination. These results confirm that the regression equation accurately represents the biological reality of the model. The total contribution of the squared terms was 11.74%, and the quadratic effect of temperature was particularly significant (F = 11.52; *p* = 0.005). However, the squared term of the weight difference was statistically insignificant (*p* = 0.829). This suggests that humidity changes are of limited importance for germination under short-term room conditions.

Regression Equation in Uncoded Units

(2) $$\mathrm{Germination}\;\mathrm{Rate}=[(41.4)-(0.10A-5.60B)-(6860A\;\times \;C)+(0.1974\;\times \;B^{2})+(317,131\;\times \;C^{2})+(392B\;\times \;C)]$$

**Figure 7:**
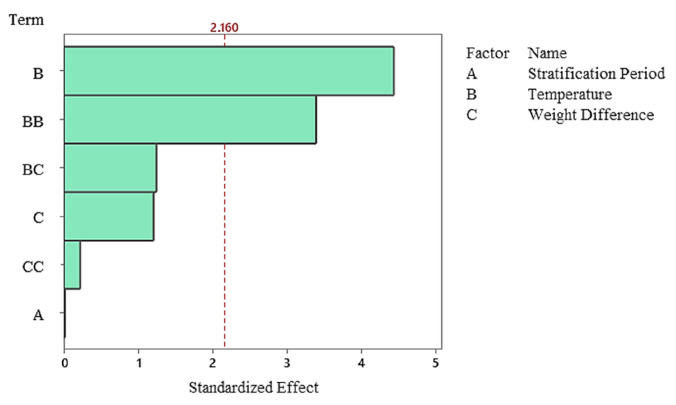
Pareto Chart of standardized effects for germination rate in the non-stratified group.

**Figure 8:**
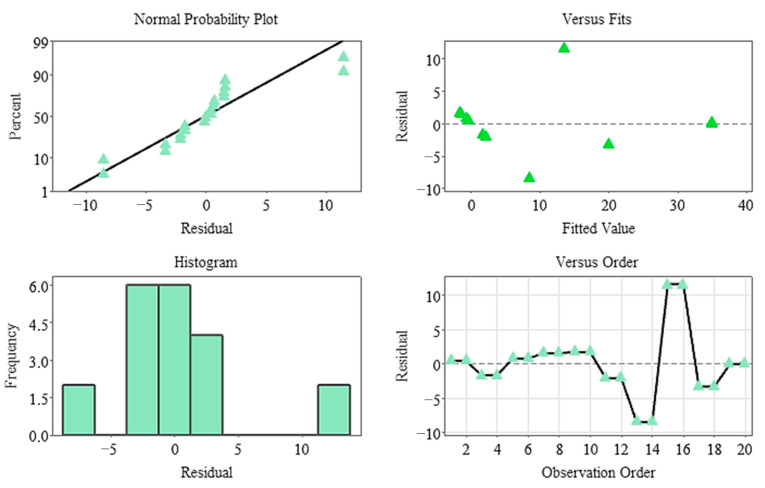
Residual plots for the germination rate model (non-stratified group).

The Pareto diagram clearly shows that the temperature factor and its quadratic effect are the strongest variables determining germination percentage. In contrast, stratification time was the lowest contributing factor in the model and remained statistically insignificant. This finding indicates that germination dynamics in the non-stratified system were almost entirely determined by temperature (Figure 7), and residual analyses indicate that the model met all assumptions. The normal probability plot yielded a linear distribution, while the histogram and Versus Fit plots revealed a homogeneous and random distribution of the residuals. No systematic deviation or time-related error was observed in the Versus Order plot (Figure 8), confirming the model’s validity and reliability. In general, it was determined that temperature was the primary factor determining germination percentage under non-stratified conditions, and this factor shaped the biological response through both linear and curvilinear effects. While the weight difference contributed minimally, stratification time had no statistically significant effect in this system.

### 3.5. RSM Findings Regarding Germination Percentage in the Stratified Group

The mathematical model developed for the stratified system showed that the experimental factors accounted for almost all of the variability observed in germination performance. The model’s coefficients of determination, adjusted, and predictive coefficients of determination were calculated as R^2^ = 99.96%, Adj-R^2^ = 99.93%, and Pred-R^2^ = 99.85%, respectively. These values indicate that the model has very high accuracy, and almost all of the explained variance is represented by the factors included in the model (Table 4). Furthermore, the overall significance level of the model was found to be extremely high (F = 2940.55; *p* < 0.001).

The total contribution of linear terms was 92.01%, with stratification time being the most dominant factor at this level (F = 7.91; *p* = 0.018). This result demonstrates that stratification directly affects germination percentage depending on time. Although the linear effect of temperature was statistically significant, its effect was quite low (0.01%), suggesting that temperature was a limited determinant compared to the dominant effect of stratification. The weight difference was also found to be linearly significant but made a small contribution (F = 16.63; *p* = 0.002). The total contribution of the squared terms was 6.59%, and all squared terms (A^2^, B^2^, and C^2^) were found to be statistically significant (*p* < 0.001).

This result indicates that the duration of cold stratification reflects the cumulative physiological processes occurring during chilling, which collectively regulate germination percentage. Rather than time itself acting as a causal agent, the observed response arises from dormancy-breaking mechanisms such as hormonal rebalancing, water uptake, and metabolic activation that progress over the stratification period. Importantly, cold stratification in seeds should not be interpreted as a passive dormant state, but rather as a physiologically active phase during which cellular, hormonal, and molecular processes progressively prepare the embryo for germination. The significance of both linear and quadratic terms demonstrates that stratification-related processes generate not only linear but also curvilinear responses in germination percentage. This pattern suggests the existence of optimal ranges for both stratification duration and temperature, beyond which the germination response tends to stabilize or plateau rather than increase further. When interaction terms were examined, it was observed that all terms A × B (F = 124.11; *p* < 0.001), A × C (F = 259.55; *p* < 0.001), and B × C (F = 242.59; *p* < 0.001) had a statistically significant effect. Although the total contribution of interactions was 1.37%, the high F-values of these terms revealed that the factors, in conjunction with each other, produced a complex germination response. This suggests that temperature and humidity changes during stratification play a joint role in reducing biophysical stress (Table 5).

The model’s second-order regression equation (Equation (3)) confirms that stratification period is the factor that most strongly shapes the germination response. The negative coefficient for the stratification period and the positive squared term in the equation indicate that germination is limited at low stratification times, increasing with increasing stratification time, but the response stabilizes after the optimum point. The positive coefficient for the temperature term and the significant squared effect indicate that temperature promotes germination depending on stratification time. The negative coefficient for the weight difference indicates that weight loss can have a negative effect on germination when it exceeds a certain threshold. The inclusion of all interaction terms in the equation and their significance demonstrates that stratification operates in conjunction with temperature and humidity factors, producing a biophysical, multifaceted mechanism.

Regression Equation in Uncoded Units

(3) $$\mathrm{Germination}\;\mathrm{Rate}=[(36.87)-(3.112\times A)+(19.37\times B)+(487\times C)-(0.1708\times A^{2})-(3.781\times B^{2})-(60,198\times C^{2})+(1.612A\times B)+(256.2A\times C)-(1234.2B\times \;C)]$$

**Figure 9:**
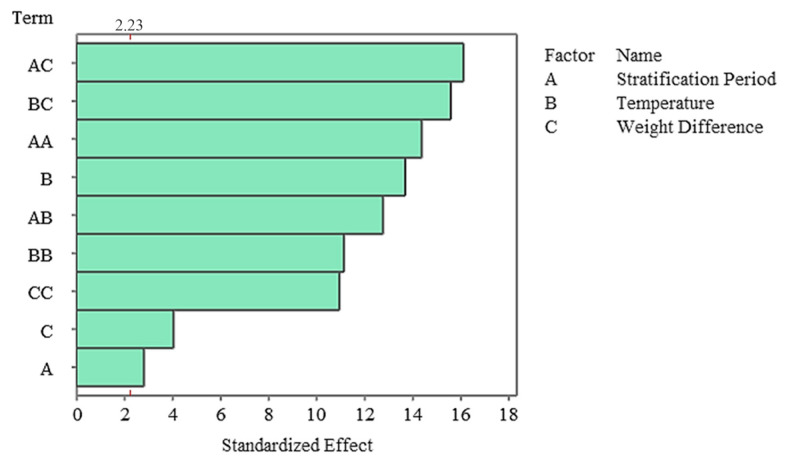
Pareto chart of standardized effects for germination rate in the stratified group.

**Figure 10:**
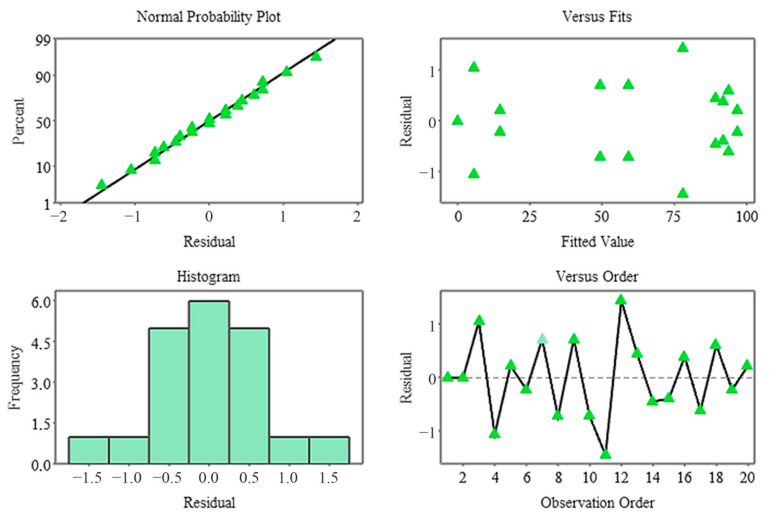
Residual plots for the germination rate model.

The Pareto diagram revealed that the strongest influences on germination percentage in the stratified system were A × C, B × C, and A2, respectively (Figure 9). Linear A, while significant, had a lesser effect overshadowed by these complex interactions. These findings demonstrate that stratification works in conjunction with biophysical conditions and has a strong germination response, particularly when coupled with moisture changes. Residual analysis revealed that the model met all assumptions, and the normal probability plot showed that the residuals followed a linear distribution. Furthermore, the histogram revealed that the residuals were symmetrical, while the Versus Fit plot confirmed homogeneity of variance and the Versus Order plot confirmed the independence of the measurements (Figure 10). The fact that all residuals remained within ±1.5 supports the high fit of the model.

In general, germination dynamics in the stratified group exhibit a highly complex structure; stratification time is the primary determinant, while temperature and weight differences work together to maximize germination. The presence of stratification makes the system sensitive to temperature and humidity changes, optimizing the germination process in a powerful and multidimensional manner.

## 4. Discussion

The results of this study clearly demonstrate that *Rudbeckia fulgida* seeds possess a pronounced physiological dormancy that requires sufficiently long cold stratification for dormancy release and successful germination. Germination percentages increased in a strong, monotonic pattern from 0% in the non-stratified control group (A) to over 96% after 135–165 days of cold treatment (J–K). This trend aligns with the fundamental role of low temperature in modulating annual dormancy cycling within temperate-zone species, as temperature regulates embryo sensitivity to secondary germination cues and drives the gradual transition from deep to shallow dormancy states [3]. The sharp increase in germination after 60 days of stratification parallels findings from related taxa in Asteraceae, where prolonged chilling promotes hormonal rebalancing, endosperm weakening, and structural remodeling necessary for radicle protrusion [8,16].

Several studies have shown that cold stratification stimulates GA biosynthesis, accelerates ABA catabolism, and activates cold-responsive transcription factors (*CBF1*–*CBF2*), thus creating a biochemical and transcriptional regulatory state conducive to germination [17]. The present findings—especially the robust increase in germination in the G–K groups (≥90 days)—agree with this hormonal framework. The strong positive correlation observed between stratification duration and germination percentage (ρ = 0.914) quantitatively supports the mechanistic model whereby extended cold exposure provides the temporal window required for full hormonal rebalancing and embryo reactivation. Similar duration-dependent stratification responses have been documented in species such as *Primula beesiana*, *Aster tripolium*, and *Pinus koraiensis*, which show maximum germination between 30 and 90 days depending on dormancy depth and ecological origin [25,26,50].

In addition to the central role of the ABA–GA hormonal balance, which regulates auxin metabolism in a localized and cascade-dependent manner, seed germination requires profound cellular reprogramming processes involving chromatin remodeling and auxin gradient re-establishment [51,52,53,54]. Recent studies emphasize that germination is initiated only after large-scale chromatin decondensation, histone modification, and transcriptional reactivation of growth-related gene networks, which enable the transition from a quiescent to a metabolically active embryonic state [4,8]. Cold stratification has been shown to promote epigenetic reprogramming by altering chromatin accessibility and facilitating the expression of genes associated with cell cycle progression, cell wall loosening, and embryonic axis elongation [16]. In parallel, the re-establishment of a functional auxin gradient is considered a critical requirement for directional growth and radicle protrusion during germination. Auxin redistribution along the embryonic axis regulates cell elongation and tissue differentiation, thereby coordinating radicle emergence and subsequent seedling establishment [4]. Cold stratification indirectly supports this process by reducing ABA-mediated repression and enabling auxin transport and signaling pathways to become operative. Thus, germination can be viewed as a two-component process comprising chromatin-level reprogramming and hormone-driven spatial patterning, both of which are progressively activated during prolonged chilling. Although auxin dynamics and chromatin modifications were not directly quantified in the present study, the strong and duration-dependent germination response observed after extended stratification provides functional evidence that these molecular and epigenetic processes were successfully completed only after sufficient chilling. This interpretation is consistent with the high germination rates obtained after 135–165 days of stratification and provides a mechanistic explanation linking prolonged chilling duration to the release of physiological dormancy in *Rudbeckia fulgida*. Seedling morphology and post-germination growth traits were not assessed in this study, as the primary objective was to quantify dormancy release and germination performance rather than subsequent developmental characteristics [55,56,57,58].

Previous work on *Rudbeckia fulgida* and related taxa has shown that seed germination, particularly in the cultivar ‘Goldsturm’, remains very low unless seeds receive a period of cold treatment, whereas some congeners germinate readily without any pretreatment [38]. This pattern is consistent with nondeep to intermediate physiological dormancy (PD) sensu This pattern aligns with characteristics of nondeep to intermediate physiological dormancy (PD), although dormancy depth has not been explicitly classified for this genus [59]. The complete absence of germination in the control group (A) and the very low germination levels in short chilling durations (B–C) in the present study support the presence of at least intermediate PD in *R. fulgida*. Although seed viability tests such as tetrazolium staining were not performed, the near-complete germination observed in long-stratified treatments (≥135 days) confirms that the seed lot was viable and that the absence of germination in early treatments reflects physiological dormancy rather than seed mortality. Seeds exhibiting intermediate PD typically require extended cold exposure (60–180 days) to transition from deep embryo quiescence to germination competence—a pattern consistent with the significant rise in germination observed after 90 days of stratification in this research. Furthermore, the minimal and irregular germination in non-stratified but long-stored groups (H2–K2) suggests that *R. fulgida* does not readily after-ripen at room temperature, which supports the notion that cold, rather than dry after-ripening, is the primary driver of dormancy release in this taxon.

Reports on *Rudbeckia* germination ecology remain limited, with prior research emphasizing habitat and population biology rather than dormancy physiology [33,34]. The current study fills this gap by providing the first comprehensive, fine-resolution (15-day interval) stratification experiment for *R. fulgida*. Literature on Asteraceae generally recommends stratification durations between 30 and 90 days; however, the exceptionally high germination rates at 135–165 days observed here indicate that *R. fulgida* may require longer chilling periods compared to other congeners. This supports earlier suggestions that stratification requirements in *Rudbeckia* vary strongly with seed source, maternal environment, and habitat-specific evolutionary pressures [38].

The strong correlations found between germination percentage, post-stratification seed weight, and temperature provide additional insights into the physiological dynamics underlying dormancy release. Weight increases after stratification likely reflect enhanced water uptake and metabolic activation, both recognized markers of embryo readiness for growth initiation [8]. The positive correlation between temperature and germination observed in both stratified and non-stratified systems is expected, as temperature governs cellular respiration, hormonal mobility, and enzymatic turnover [4]. However, only in the stratified system do these temperature effects translate into high germination, reinforcing that cold exposure is the principal factor unlocking germination competence.

The application of RSM allowed the multidimensional structure of the germination process to be quantitatively modeled, capturing nonlinear relationships, curvature, and interaction effects that traditional unifactorial statistical approaches cannot fully represent. The model’s exceptionally high predictive accuracy (99%) indicates that the key variables measured—stratification length, temperature, and weight change—collectively explain the germination response with high precision. Biologically, this means that germination in *R. fulgida* is governed by an integrated response network in which chilling duration provides the essential dormancy-breaking stimulus, while temperature and post-imbibition physiological changes modulate the efficiency and speed of the germination process [60,61,62]. Such multidimensional modeling has been advocated in recent studies addressing rooting efficiency, microclimate–plant interactions, and plant–material behavior under experimental design frameworks [29,31,63,64]. While the present study did not simulate fluctuating field conditions such as diurnal temperature or humidity cycles and soil pressure, its primary objective was to establish a standardized dormancy-breaking protocol and a robust predictive model under controlled conditions. This controlled approach allows the isolation of key drivers of germination and provides a reproducible framework that can later be integrated with field-based ecological studies.

From a practical standpoint, these findings provide actionable guidance for nursery propagation, restoration programs, and landscape applications. The identification of 135–165 days as the optimal chilling range offers a clear protocol for large-scale seedling production of *R. fulgida*, a species valued for ornamental use, drought tolerance, pollinator support, and ecological restoration. In natural habitats, such extended chilling requirements suggest that *R. fulgida* is tightly adapted to temperate winter dynamics, and its persistence may depend on reliable cold-season cues. This has implications under climate change scenarios, where warming winters may disrupt natural dormancy-cycling mechanisms [10,65,66,67], potentially affecting recruitment success. The demonstrated capacity of RSM to model germination responses accurately also provides a transferable framework for optimizing stratification protocols in other native and horticulturally important species.

## 5. Conclusions

This study presents a high-resolution evaluation of cold stratification requirements in *Rudbeckia fulgida* Aiton using eleven chilling durations applied at 15-day intervals. The results clearly demonstrate that the species exhibits physiological dormancy and that successful germination is achieved only through prolonged cold stratification. Seeds maintained at room temperature showed either no germination or irregular and low germination responses, whereas the highest germination rates (96–100%) were obtained after 135–165 days of chilling, identifying this range as optimal.

Correlation analyses confirmed strong positive relationships between stratification duration, germination percentage, temperature, and post-stratification seed weight, indicating that extended chilling promotes dormancy-breaking physiological processes such as water uptake and metabolic activation. In contrast, time alone without cold exposure was insufficient to induce consistent germination.

The application of Response Surface Methodology (RSM) provided a highly accurate predictive model (99%), successfully capturing the nonlinear and interactive effects of stratification duration, temperature, and seed physiological traits. These findings offer practical guidance for nursery production and restoration practices, recommending a minimum of 135 days of cold stratification for reliable seed propagation. Additionally, the strong dependence on prolonged chilling highlights the potential sensitivity of *R. fulgida* germination to future changes in winter temperature regimes.

## Figures and Tables

**Table 1:** Description of treatment codes used in the study.

Treatment Code	Treatment Duration (Days)	Condition
A	0	Cold stratification
B	15	Cold stratification
C	30	Cold stratification
D	45	Cold stratification
E	60	Cold stratification
F	75	Cold stratification
G	90	Cold stratification
H	105	Cold stratification
I	120	Cold stratification
J	135	Cold stratification
K	165	Cold stratification
A2	0	Room temperature (non-stratified)
B2	15	Room temperature (non-stratified)
C2	30	Room temperature (non-stratified)
D2	45	Room temperature (non-stratified)
E2	60	Room temperature (non-stratified)
F2	75	Room temperature (non-stratified)
G2	90	Room temperature (non-stratified)
H2	105	Room temperature (non-stratified)
I2	120	Room temperature (non-stratified)
J2	135	Room temperature (non-stratified)
K2	165	Room temperature (non-stratified)

**Table 2:** Model Summary.

S	R-sq	R-sq (Adj)	PRESS	R-sq (Pred)	AICc	BIC
5.95306	85.04%	78.14%	-	-	148.59	143.46

**Table 3:** Analysis of Variance.

Source	DF	Seq SS	Contribution	Adj SS	Adj MS	F-Value	*p*-Value
Model	6	2619.41	85.04%	2619.41	436.569	12.32	0.000
Linear	3	2202.84	71.52%	914.83	304.944	8.60	0.002
A	1	786.41	25.53%	0.01	0.006	0.00	0.990
B	1	1292.83	41.97%	697.52	697.524	19.68	0.001
C	1	123.60	4.01%	51.70	51.701	1.46	0.249
Square	2	361.51	11.74%	408.35	204.176	5.76	0.016
B^2^	1	318.28	10.33%	408.18	408.178	11.52	0.005
C^2^	1	43.23	1.40%	1.72	1.725	0.05	0.829
2-Way Interaction	1	55.06	1.79%	55.06	55.063	1.55	0.235
B × C	1	55.06	1.79%	55.06	55.063	1.55	0.235
Error	13	460.71	14.96%	460.71	35.439		
Lack-of-Fit	4	460.71	14.96%	460.71	115.177	-	-
Pure Error	9	0.00	0.00%	0.00	0.000		
Total	19	3080.12	100.00%				

**Table 4:** Model Summary.

S	R-sq	R-sq (Adj)	PRESS	R-sq (Pred)	AICc	BIC
1.00704	99.96%	99.93%	40.5649	99.85%	98.18	76.13

**Table 5:** Analysis of Variance.

Source	DF	Seq SS	Contribution	Adj SS	Adj MS	F-Value	*p*-Value
Model	9	26,838.7	99.96%	26,838.7	2982.07	2940.55	0.000
Linear	3	24,702.6	92.01%	5894.6	1964.87	1937.51	0.000
A	1	24,642.4	91.78%	8.0	8.03	7.91	0.018
B	1	1.5	0.01%	189.7	189.69	187.05	0.000
C	1	58.7	0.22%	16.7	16.66	16.43	0.002
Square	3	1769.2	6.59%	506.0	168.66	166.32	0.000
A^2^	1	1572.6	5.86%	209.5	209.49	206.57	0.000
B^2^	1	16.6	0.06%	125.9	125.86	124.11	0.000
C^2^	1	180.0	0.67%	121.6	121.62	119.92	0.000
2-Way Interaction	3	366.9	1.37%	366.9	122.29	120.59	0.000
A × B	1	98.8	0.37%	165.1	165.06	162.76	0.000
A × C	1	22.1	0.08%	263.2	263.22	259.55	0.000
B × C	1	246.0	0.92%	246.0	246.02	242.59	0.000
Error	10	10.1	0.04%	10.1	1.01		
Total	19	26,848.8	100.00%				

## Data Availability

The original contributions presented in the study are included in the article. Further inquiries can be directed to the corresponding author.

## Abbreviations

The following abbreviations are used in this manuscript:

ABA	Abscisic Acid
BC	Interaction term between temperature (B) and weight difference (C) in RSM model
BRs	Brassinosteroid
*CBF1/CBF2*	C-repeat Binding Factors 1 and 2 (cold-responsive transcription factors)
*DOG1*	Delay of Germination 1 gene
DF	Degrees of Freedom
GA	Gibberellin
H	Kruskal–Wallis test statistic
N	Sample Size
PD	Physiological Dormancy
Pred-R^2^	Predictive Coefficient of Determination
ROS	Reactive Oxygen Species
RSM	Response Surface Methodology
Seq SS	Sequential Sum of Squares
SPSS	Statistical Package for the Social Sciences
ρ	Spearman Rank Correlation Coefficient
°C	Degrees Celsius
